# Glycosylation defects, offset by PEPCK-M, drive entosis in breast carcinoma cells

**DOI:** 10.1038/s41419-022-05177-x

**Published:** 2022-08-24

**Authors:** Petra Hyroššová, Marc Aragó, Cristina Muñoz-Pinedo, Francesc Viñals, Pablo M. García-Rovés, Carmen Escolano, Andrés Méndez-Lucas, Jose C. Perales

**Affiliations:** 1 Department of Physiological Sciences, School of Medicine, University of Barcelona-IDIBELL, L’Hospitalet de Llobregat, Spain; 2 Programs of Molecular Mechanisms and Experimental Therapeutics in Oncology (Oncobell), and Cancer Therapeutics Resistance (ProCURE), Catalan Institute of Oncology, Bellvitge Institute for Biomedical Research (IDIBELL), L’Hospitalet del Llobregat, Spain; 3 Laboratory of Medicinal Chemistry (Associated Unit to CSIC), Faculty of Pharmacy and Food Sciences, and Institute of Biomedicine (IBUB), University of Barcelona, Barcelona, Spain

**Keywords:** Entosis, Cancer metabolism

## Abstract

On glucose restriction, epithelial cells can undergo entosis, a cell-in-cell cannibalistic process, to allow considerable withstanding to this metabolic stress. Thus, we hypothesized that reduced protein glycosylation might participate in the activation of this cell survival pathway. Glucose deprivation promoted entosis in an MCF7 breast carcinoma model, as evaluated by direct inspection under the microscope, or revealed by a shift to apoptosis + necrosis in cells undergoing entosis treated with a Rho-GTPase kinase inhibitor (ROCKi). In this context, curbing protein glycosylation defects with N-acetyl-glucosamine partially rescued entosis, whereas limiting glycosylation in the presence of glucose with tunicamycin or NGI-1, but not with other unrelated ER-stress inducers such as thapsigargin or amino-acid limitation, stimulated entosis. Mitochondrial phosphoenolpyruvate carboxykinase (PEPCK-M; PCK2) is upregulated by glucose deprivation, thereby enhancing cell survival. Therefore, we presumed that PEPCK-M could play a role in this process by offsetting key metabolites into glycosyl moieties using alternative substrates. PEPCK-M inhibition using iPEPCK-2 promoted entosis in the absence of glucose, whereas its overexpression inhibited entosis. PEPCK-M inhibition had a direct role on total protein glycosylation as determined by Concanavalin A binding, and the specific ratio of fully glycosylated LAMP1 or E-cadherin. The content of metabolites, and the fluxes from ^13^C-glutamine label into glycolytic intermediates up to glucose-6-phosphate, and ribose- and ribulose-5-phosphate, was dependent on PEPCK-M content as measured by GC/MS. All in all, we demonstrate for the first time that protein glycosylation defects precede and initiate the entosis process and implicates PEPCK-M in this survival program to dampen the consequences of glucose deprivation. These results have broad implications to our understanding of tumor metabolism and treatment strategies.

## Introduction

Nutritional stress impairs cell growth in tumor cells of epithelial origin by activating a novel cell survival process termed entosis which is characterized by *cell-in-cell* invasion of neighboring cells destined for non-apoptotic cell death [1]. Entosis is initiated in conditions of glucose limitation, cell matrix detachment or during mitosis in very specific settings [1, 2, 3]. However, it is not clear what the sensory mechanism is for the initiation of the program; and although the E-cadherin/P-cadherin family of proteins are required for the process to fully initiate after cell detachment, the triggering mechanism for the activation of the cell-in-cell invasion process in an E-cadherin dependent manner upon glucose limitation has not been reported. Starving of glucose carbon has been shown to activate a canonical ER-stress response, but also to activate a signaling cascade initiated by the phosphorylation of AMP-K [3], the latter step being required for the process to complete effectively. These studies suggest a link between an energy/metabolic deficit and the activation of entosis. In this context, we hypothesize that protein glycosylation alterations might initiate this cascade, that would feedback into the upregulation of several ER-stress target genes that are required to compensate the metabolic derangements induced by glucose deprivation.

We have recently identified the mitochondrial isoform of phosphoenolpyruvate carboxykinase (PEPCK-M; PCK2) as a target gene for ATF4, the master regulator of ER- and amino-acid stress responses [4]. PEPCK-M was upregulated by effectors of this pathway by recruiting ATF4 to a consensus AARE site located at the PEPCK-M proximal promoter. In these conditions, PEPCK-M activity is necessary to tip the balance of the cell towards cell survival in a model of human breast carcinoma (MCF7). The importance of chronic ER-stress to induce adaptive responses in cancer cells in vivo suggested that the pathway might be relevant to cancer cell metabolism and progression.

PEPCK-M role on cataplerosis and TCA-cycle flux regulation has been demonstrated in the liver [5]. In addition, PEPCK-M activity has been shown to promote the recycling of GTP from the succinyl CoA synthetase pathway to stimulate TCA-cycle flux in pancreatic β-cells [6]. However, the metabolic role of this enzyme in cancer cells, and in other non-gluconeogenic cell types, remains elusive.

Consistent with this view, others have demonstrated the relevance of PEPCK-M in cell growth and chemo resistance in lung and colon cancer cells, respectively [7, 8]. In every model, PEPCK-M seems to help tumor cell growth and survival. Both the mechanism for PEPCK-M related effects on cancer cell growth and the way PEPCK-M fluxes interact with tumor metabolism to promote survival will serve to identify the potential of this pathway as a target for therapeutic intervention in cancer and is the focus of the present work.

We describe here a mechanism for the initiation of entosis whereby protein glycosylation defects are responsible for the induction of the pathway even in the presence of glucose. Therefore, a bioenergetic stress sensor or the canonical ER-stress pathway are not sufficient to induce the pathway. A role for PEPCK-M in modulating the entry into the entosis cell survival program in conditions of glucose deprivation is also shown. PEPCK-M activity rescues, and its downregulation exacerbates, metabolic stress sensor pathways by contributing direct fluxes to glycolytic intermediate pools above PEP, therefore sustaining the formation of glycosyl moieties for protein glycosylation that keep entosis in check.

## Materials and methods

### Cell culture

Wild-type and gene modified clones of the MCF7 human breast carcinoma cell line were maintained in Dulbecco modified Eagle’s medium (DMEM) supplemented with 10% Fetal Bovine Serum, 2 mM L-glutamine, 10000 units/ml penicillin, 10 mg/ml streptomycin (all from Biological Industries, Beit HaEmek, Israel), and cultured at 37 °C in a humidified incubator supplied with 5% CO2. Glucose free medium was supplemented with 4 mM L-Glutamine, 10% Fetal Bovine Serum, 10000 units/ml penicillin and 10 mg/ml streptomycin. For short term experiments a dialyzed Fetal Bovine Serum (Biological Industries) was used. ROCK inhibitor Y27632 (ROCKi) was purchased from MedChemExpress (Monmouth Junction, NJ, USA) and used at a final concentration of 10 µM. PEPCK-M inhibitor (iPEPCK-2) was produced in the laboratory of Dr. Carmen Escolano and used at a final concentration of 5 µM. Inhibitors of glycosylation, NGI-1 and Tunicamycin used at final concentration 10 µM and 3 µg/ml, respectively, were purchased from Sigma-Aldrich (St. Louis, MO, USA). Thapsigargin was purchased from Sigma-Aldrich (St. Louis, MO, USA) and used at a final concentration 0.1 µM. Taxol (Teva, Jerusalem, Israel) was used at final concentration 1 µM.

### Transduction

Protocols were performed as recommended by manufacturer. For overexpression of PCK2, MCF7 cells were infected with a PCK2 Human ORFeome lentiviral particles (GeneCopoeia, Rockville, MD, USA; clone ID: LP-OL06695-LX304-0200-S) and denominated L-PCK2. Cells were selected with 2 μg/ml blasticidin for 1 week.

### Establishment of ATF4 knockout MCF7 cell line with CRISPR/Cas9 system

To generate a pool of MCF7 cells lacking ATF4 (ATF4^KO^), guide RNAs (gRNA) were designed to target PCK2 using an online gRNA design tool (CHOPCHOP; https://chopchop.cbu.uib.no), and synthesized, annealed and cloned into the pSpCas9(BB)-2A-puro vector (Adgene, Watertown, MA, USA) as described (ZhangLab; https://media.addgene.org/cms/files/Zhang_lab_LentiCRISPR_library_protocol.pdf). Twenty-four hours posttransfection, puromycin was added for 24 h at 2 μg/ml for selection and pool of selected cells was tested for protein knockdown by western blot.

### Cell death analysis

Apoptosis and necrosis were quantified by flow cytometry (FACS Calibur, BD Biosciences, Mountain View, CA, USA) using eBioscience™ Annexin V-APC and SYTOX™ Green Nucleic Acid Stain (Invitrogen, Carlsbad, CA, USA) following the manufacturer’s instructions. Cells stained by Annexin V-APC, SYTOX™ or co-stained by both has been identified as population of apoptotic and necrotic cells.

### Western blot

Proteins were extracted by using RIPA buffer supplemented with protease and phosphatase inhibitors. Equal quantity of protein per lane was resolved by SDS-PAGE gel and transferred onto PVDF membranes.

For classic protein detection, blots were blocked in 5% skimmed milk in 0.1% Tween Tris-buffered saline for 1 h at room temperature (RT), and then incubated overnight with primary antibody at 4 °C. The following primary antibodies were used: anti-LAMP1 (ab62562; Abcam, Cambridge, MA, USA), anti-E-Cadherin (24E10; Cell Signaling Technologies, Danvers, MA, USA), anti-ATF4 (sc200; Santa Cruz Biotechnology, Santa Cruz, CA, USA), anti-p-eIF2α (sc101670; Santa Cruz Biotechnology, Santa Cruz, Ca, USA), anti-γ tubulin (T6557, Sigma-Aldrich, St. Louis, MO, USA). Afterwards, blots were incubated with horseradish peroxidase-conjugated secondary antibodies anti-Mouse and anti-Rabbit (Advansta, San Jose, CA, USA) reconstituted in 0.1% Tween Tris-buffered saline with 5% skimmed milk for 1 h at room temperature.

For lectin detection, blots were blocked in 0.5% Tween Phosphate-buffer saline for 1 h at RT and then incubated 1 h at RT with anti-Concanavalin A (Sigma-Aldrich, St. Louis, MO, USA) 1.5 µg/ml in blocking solution. Afterwards, blots were incubated with Streptavidine-HRP (Abcam, Cambridge, MA, USA) 1:3000 in blocking solution.

Horseradish peroxidase activity was detected with ECL Pierce substrate (Thermo Fisher Scientific, Waltham, MA, USA) by a chemiluminescent detection system (FujifilmLAS 3000 Intelligent Dark Box IV imaging system).

### Metabolomics

For labeling studies, cells were plated in 6-well plates at 0.25 × 106 cells per well and allowed to adhere overnight. Cells were washed abundantly with PBS and treated with medium lacking glucose for 72 h. For last 9 h of the experiment, glucose free media with dialyzed FCS containing 4 mM U-^13^C labeled glutamine was added. Control cells were grown in medium containing 25 mM glucose for 48 h. At the end of the experiment, cells were washed twice with cold PBS and harvested using 900 µl of cold methanol/chloroform (2:1, v:v). Scyllo-inositol and L-Norleucine (3 nmol) were used as internal standards. GC/MS analysis of polar metabolites was performed as previously described [9]. Briefly, a part of the polar fraction was washed twice with methanol, derivatized by methoximation (Sigma, 20 µl, 20 mg/ml in pyridine) and trimethylsilylation (20 µl of N,O-bis(trimethylsilyl)trifluoroacetamide reagent (BSTFA) containing 1% trimethylchlorosilane (TMCS), Supelco). Analyses were performed in 5975 MSD with 6890 N and 5975 C MSD with 7890 A GC-MS system (Agilent). Splitless injection (injection temperature 270 °C) onto a 30 m + 10 m × 0.25 mm DB-5MS + DG column (Agilent J&W) was used, using helium as the carrier gas, in electron ionization (EI) mode. The initial oven temperature was 70 °C (2 min), followed by temperature gradients to 295 °C at 12.5 °C/min and then to 320 °C 25 °C/min (held for 3 min). Metabolite quantification and isotopologue distributions were corrected for the occurrence of natural isotopes in both the metabolite and the derivatization reagent. MassHunter Quantitative Analysis software (B.06.00 SP01, Agilent Technologies) was used for data analysis and peak quantifications. The level of labeling of individual metabolites was corrected for natural abundance of isotopes in both the metabolite and the derivatization reagent, and abundances were calculated by comparison to responses of known amounts of authentic standards.Labeled glutamine contributed close to 100% of the TCA-cycle metabolites isotopologues after incubation as shown in Supplementary Fig. 2.

### Metabolite extraction for PEP and G6P assay

1.75 × 10^6^ cells were plated in 10 cm petri dish and allowed to adhere overnight. Cells were washed with PBS and treated with medium supplemented with dialyzed FCS and containing 0 mM or 25 mM glucose, respectively. After 24 h, metabolites were extracted using perchloric acid (1 M) and neutralized to pH 7–8 using 3 M KHCO_3_.

### PEP determination assay

PEP was measured by using enzymatic assay catalyzed by pyruvate kinase. Per sample, 10 µl of metabolite extract was mixed with 10 µl of reconstituted enzyme from StayBrite^TM^ (Biovision, Milipitas, CA, USA) and filled up to 100 µl with buffer (final concentration in 100 µl of assay volume: Gly-Gly 50 mM (pH 7), KCl 0.1 M, MgCl_2_ 5 mM and MgADP 1 mM). The amount of ATP formed during the conversion of PEP to pyruvate was measured as an increment in luminescence and measured with a luminometer (TD 20/20; Turner Designs, San Jose, CA, USA) 2 min after the addition of 10 µl of pyruvate kinase (13.5 U/mL) per sample. The results were corrected for background luminescence and normalized by protein content.

### G6P determination assay

G6P was measured by using enzymatic assay catalyzed by Glucose-6-phosphate dehydrogenase, where NADPH formation is proportional to G6P amount. Per sample, 100 µl of metabolite extract was mixed with 90 µl of buffer (final concentration in 200 µl of assay volume: Gly-Gly 50 mM (pH 7), KCl 0.1 M, MgCl_2_ 5 mM and NADP 1 mM). After reading background, 10 µl of Glucose-6-phosphate dehydrogenase (18.2 U/ml) was added. The amount of formed NADPH was determined by measuring fluorescence (excitation 340 nm/emission 460 nm) using the Fluostar Optima BMG Labtech system.

### RNA extraction and quantitative Real-Time PCR

Total RNA was extracted using TRIsure (Bioline, London, UK) and cDNA was synthesized from 2 ug of RNA using High-Capacity cDNA Reverse Transcription kit (Invitrogen, Carlsbad, CA, USA). Quantitative real-time PCR was performed using SensiFAST™ Probe Hi-ROX Kit (Bioline) on TaqMan 7900HT real-time RT-PCR system (Applied Biosystems, Foster City, CA). The PCR conditions were 95 °C for 10 min, and 40 cycles of 95 °C for 15 s and 60 °C for 30 s. Data analysis is based on the ΔΔCt method, where cycle threshold (Ct) values of genes of interest were normalized to Ct values of housekeeping gene (TBP2 or GUSB) and then to control group.

### Identification of entotic cells

Cells (0.05-0.1 × 10^6^ cells/well) were seeded in µ-Slide 8 Well dish in complete media. Next day, cells were treated with glucose deprivation medium in the presence of vehicle, iPEPCK-2 (5 µM) or N-acetyl glucosamine (NAG, 15 mM). After 72 h, nuclei were stained with Hoechst 33342 at a concentration 100 ng/ml and the number of entotic structures per total number of cells was counted. Similarly, we evaluated the presence of entotic structures in media containing glucose. Cells were seeded in complete media at a concentration of 0.025-0.05 × 10^6^ cells/well in µ-Slide 8 Well dish. Next day media was changed. Cells were treated for 48 h with complete media containing tunicamycin (TUN, 3 µg/ml), NGI-1 (10 µM) or vehicle, and for 24 h with complete media containing taxol (1 µM), thapsigargin (0.1 µM) or vehicle. In the case of amino-acid deprivation, cells were treated for 48 h with DMEM media lacking arginine, lysine, serine and glycine and supplemented with dialyzed FCS. Afterwards, nuclei were stained with Hoechst 33342 at a concentration 100 ng/ml and the number of entotic structures per total number of cells was counted. Samples were imaged using a Zeiss Axioplan I fluorescent microscope (Carl Zeiss, Oberkochen, Germany). Entosis was identified as observation of cell-in-cell structures.

### Fluorescent microscopy

0.05 × 10^6^ cells were seeded on poly-L-lysine coated coverslips (Ø 12 mm) in 24 well plate. Cells were fixed with 4% PFA for 10 min at RT and washed 3 times with PBS. Cells were blocked in 2% NHS with 0.025% Triton in PBS for 1 h and incubated overnight with primary antibody at 4 °C. Following primary antibodies were used: anti-E-cadherin, anti-β-catenin (BD Biosciences, Franklin Lakes, NJ, USA and anti-LAMP1 (Abcam)). Samples were than incubated with secondary antibodies Alexa Flour 633 anti-Mouse (Thermo Fisher Scientific, Waltham, MA, USA) and Alexa Flour 488 and counterstained with Hoechst 33342. Samples were imaged using a Zeiss Axioplan I fluorescent microscope (Carl Zeiss, Oberkochen, Germany).

### Tissue microarray

Panel (BCN962, Biomax, Rockville, MD, USA) containing multiple organ carcinoma and adjacent normal tissue was deparaffinized and rehydrated according to standard procedures. Antigen retrieval was performed by heating the slide in 10 mM sodium citrate buffer (pH 6) in a pressure cooker. The highest pressure was maintained for 3 min, and samples were let to cool down for 20 min. Endogenous peroxidase activity was inactivated by incubating samples in 6% H_2_O_2_ for 15 min. Samples were blocked with 20% goat serum in PBS and then incubated O/N with primary antibody against PEPCK-M (ab70359, Abcam) and peroxidase-based secondary anti-goat antibody. Antigen-antibody complexes were detected with a DAB peroxidase substrate kit (Dako Agilent, Santa Clara, CA, USA) according to the manufacturer’s protocol. Samples were counterstained with hematoxylin, dehydrated, and mounted with DPX. Preparations were visualized, and images were captured with Nikon Eclipse 800 light microscope (Nikon, Tokyo, Japan).

### Statistical analysis

Results are expressed as the means ± SEM. Statistical analysis was performed by one-way ANOVA (Newman–Keuls post-hoc test) and paired, when assessing the effects on independent experimental replicates, or unpaired, when assessing the effects on biological replicates, two-tailed Student’s *t*-test, using GraphPad Prism® software. *P* < 0.05 was considered significant.

Sample size was chosen in each experiment to ensure adequate power to identify significant differences at a *P* < 0.005 among groups using standard statistical tests, either 2-tailed Student’s *t*-test when comparing paired groups of experimental sets, or Newman–Keus post-hoc tests on unpaired one-way ANOVA analyses when testing an effect among a group of variables. Graphpad software was utilized for minimum sample size requirements, although most experiments provided a larger sample size than the minimum required.

Normal distribution and variation estimates were assessed in Graphpad software for each dataset before analysis of statistical significance, as required to be appropriate for the statistical tests utilized. Variance was similar among the groups compared.

## Results

### Entosis is promoted by insufficient protein glycosylation

**Fig. 1:**
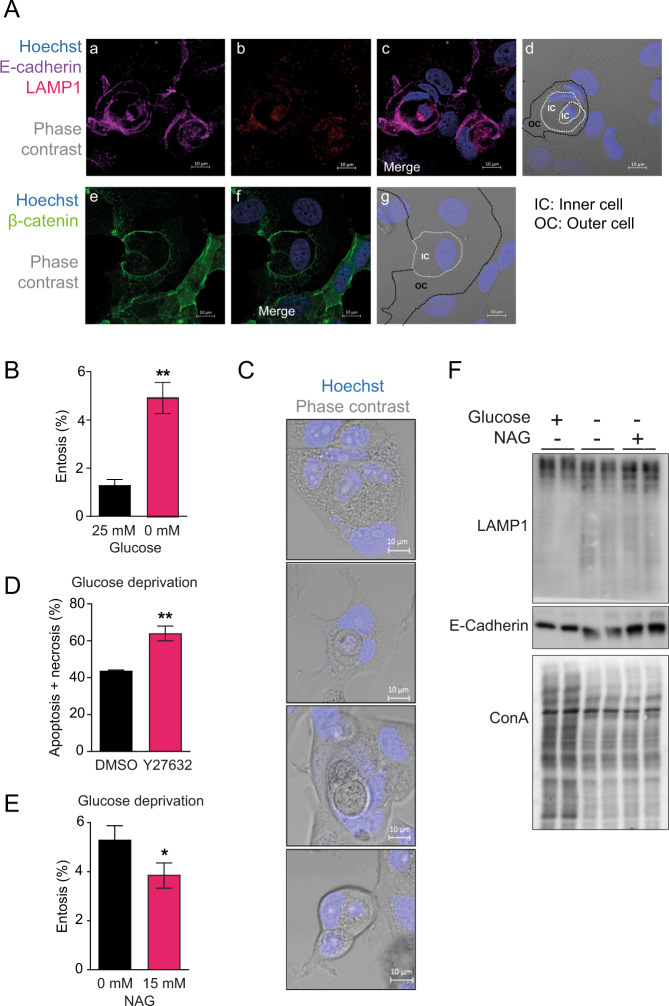
Entosis in MCF7 breast epithelial cells after glucose deprivation. **A** Confocal images of entosis events in wild-type MCF7 cells grown for 72 h in 0 mM glucose media. Cells were stained with antibodies against E-cadherin (violet), LAMP1 (red) and β-catenin (green). Nuclei were stained with Hoechst (blue). **B** Percentage of entosis in wild-type MCF7 cells after manual counting of cell-in-cell bodies representing entotic/engulfed cells at the 72 h time point at 0 mM glucose. Entotic cells were determined using confocal imaging (Two-tailed *t*-test; *n* = 5). **C** Representative confocal photographs of engulfed cell-in-cell bodies identified as entotic bodies for manual counting. Nuclei were stained with Hoechst (blue). **D** Percentage of apoptotic+necrotic cells in wild-type MCF7 cells after 72 h of glucose deprivation in the presence of ROCKi (Y27632, 10 µM) or vehicle (DMSO). Cells were analyzed by flow cytometry using Annexin V-APC and SYTOX™ Green Nucleic Acid Stain (Two-tailed *t*-test; *n* = 3). **E** Percentage of entotitic cells observed in wild-type MCF7 cells at the 72 h time point of growth in 0 mM glucose media in the presence or absence of NAG (15 mM). Entotic cells were determined using confocal imaging and manually counted (Two-tailed *t*-test; *n* = 3). **F** Western blot for human LAMP1, E-cadherin and Concanavalin A after culturing the cells at 0 mM glucose, or 0 mM glucose in the presence of NAG (15 mM).

We have used a glucose deprivation MCF7 breast carcinoma cell model to investigate the mechanisms involved in entosis. These cells present E-cadherin and β-catenin positive membrane enclosures with LAMP1 expression engulfing Hoechst positive nuclei structures when incubated in the absence of glucose, compatible with *cell-in-cell* invasion which are characteristic of the entotic process (Fig. 1A). Quantification of micrographs from 72 h cultured MCF7 deprived of glucose, showed about 5% of the cells undergoing active entosis (Fig. 1B and representative images in Fig. 1C). To assay entosis indirectly in a more quantitative and sensitive manner, we took advantage of Y27632, an inhibitor of Rho-associated protein kinase (ROCK), that blocks the initiation phase of the entosis program and pushes the cell into apoptosis+necrosis [10]. Y27632 treatment of wild-type MCF7 cells resulted in increased amounts of AnnexinV+7AAD positive cells (apoptotic + necrotic population; Fig. 1D) as measured by flow cytometry. The observed increment in apoptotic+necrotic cell population, ~12%, was higher than the entotic events previously quantitated under the microscope (Fig. 1B and representative images in Fig. 1C), as this strategy should provide accumulated cell counts over the whole treatment period (72 h).

We hypothesized that impaired protein glycosylation due to limiting glucose carbon supply has a role in the initiation of the entosis program. Therefore, we assessed whether entosis was abrogated by rescuing protein glycosylation defects with N-acetyl-glucosamine (NAG) supplementation. NAG reduced the percentage of entotic structures observed under the microscope (Fig. 1E). Protein glycosylation was reduced by glucose deprivation and partially rescued by NAG, as indirectly evaluated by the relative amount of full size versus intermediate, smeared bands, indicative of partially glycosylated protein for specific membrane protein markers such as LAMP1 (Fig. 1F; top panel), or E-Cadherin (Fig. 1F; middle panel). NAG did not significantly rescue Concanavalin A binding probably due to Concanavalin A binding limitations to partially glycosylated proteins (Fig. 1F; bottom panel).

**Fig. 2:**
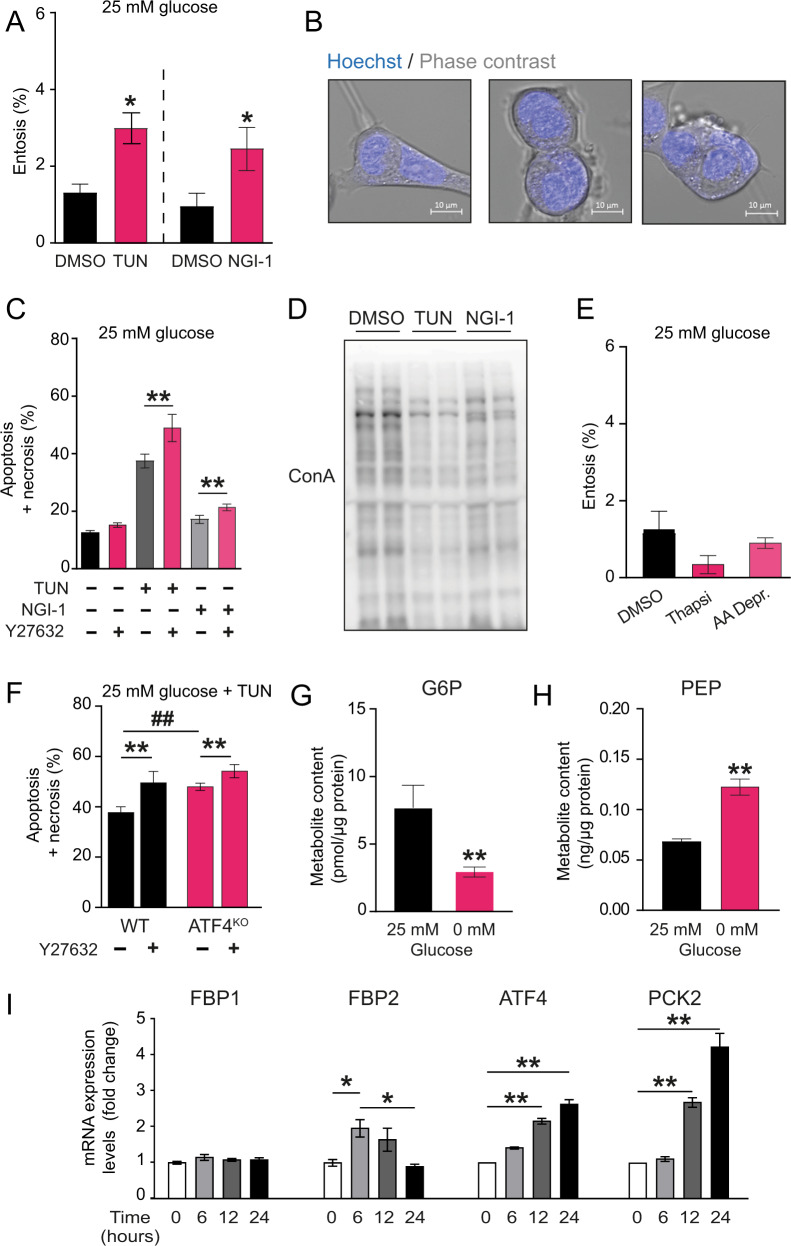
Glycosylation defects induce entosis in MCF7 cells in an ER-stress independent manner. **A** Percentage of manually counted entotic cells observed in wild-type MCF7 cells after 48 h of growth in high glucose media in the presence of tunicamycin (TUN, 3 µg/ml), NGI-1 (10 µM), or vehicle (DMSO) (Two-tailed *t*-test; *n* = 3). **B** Representative confocal photographs of engulfed cell-in-cell bodies qualified as entotic for manual counting. Nuclei were stained with Hoechst (blue). **C** Percentage of apoptotic+necrotic wild-type MCF7 cells detected after 48 h growth in high glucose media in the presence of tunicamycin (TUN, 3 µg/ml), NGI-1 (10 µM), Y27632 (10 µM) or vehicle (DMSO). Cells were analyzed by flow cytometry using Annexin V-APC and SYTOX™ Green Nucleic Acid Stain. Statistical analysis queried for TUN or NGI-1 treated with Y27632 versus DMSO (Two-tailed *t*-test; *n* = 3). **D** Western blot for Concanavalin A developed proteins, after culturing the cells in high glucose media in the presence of tunicamycin (TUN, 3 µg/ml), NGI-1 (10 µM) or vehicle (DMSO). **E** Percentage of entosis observed in wild-type MCF7 cells after growth in high glucose media in the presence of thapsigargin (100 nM; 24 h), in amino-acid limitation media (AA Depr, DMEM media w/o serine, glycin, arginin and leucine; 48 h), or vehicle (DMSO). Statistical analysis queried for AA Depr or Thapsi treated versus DMSO control (Two-tailed *t*-test; *n* = 4). **F** Percentage of apoptotic+necrotic cells observed after 48 h of growth in high glucose media in the presence of tunicamycin (TUN, 3 µg/ml) after treatment with Y27632 (10 µM) or vehicle (DMSO) in either wild-type MCF7 cells or ATF4^KO^ cells. Cells were analyzed by flow cytometry using Annexin V-APC and SYTOX™ Green Nucleic Acid Stain. Statistical analysis queried ATF4^KO^ versus WT (#, One-way ANOVA with Newman–Keuls post-hoc test; *n* = 4) or Y27632 versus DMSO (*, Two-tailed *t*-test; *n* = 4). **G** Concentration of G6P in wild-type MCF7 cell extracts after 24 h of growth in the presence of 25 mM glucose and in 0 mM glucose. Concentration was measured by using enzymatic assay and values were normalized by protein content (Two-tailed *t*-test; *n* = 3). **H** Concentration of PEP in wild-type MCF7 cell extracts after 24 h of growth in the presence of 25 mM glucose and in 0 mM glucose. Concentration was measured by using enzymatic assay and values were normalized by protein content (Two-tailed *t*-test; *n* = 3). **I** Relative gene expression of FBP1, FBP2, ATF4 and PCK2 at various times after glucose deprivation. qRT-PCR was performed on total RNA extracts from wild-type MCF7 cells in culture at various time points (One-way ANOVA with Newman–Keuls post-hoc test; *n* = 3).

Conversely, we speculated that inhibiting protein glycosylation (using tunicamycin or NGI-1) in the presence of glucose was sufficient to initiate entosis. Tunicamycin and NGI-1 activated the canonical ER-stress response as expected in these conditions (Supplementary Fig. 1A, B), and each protein glycosylation inhibitor was able to induce entosis in wild-type MCF7 cells in the presence of glucose as a substantial number of entotic bodies could be measured under the microscope (Fig. 2A and representative images in Fig. 2B). Consistently, inhibition of entosis using ROCKi increased the population of apoptotic+necrotic cells (Fig. 2C).

Total protein glycosylation was reduced upon tunicamycin or NGI-1 treatment as determined by Concanavalin A western blot (Fig. 2D). Also, the relative amount of intermediate, smeared bands for specific membrane protein markers such as LAMP1 was increased in the presence of tunicamycin (Supplementary Fig. 1B), all-in-all consistent with defects in glycosylation resulting in the appearance of partially glycosylated peptides.

As either glucose deprivation or tunicamycin/NGI-1 induced ER-stress, we next evaluated whether entosis was a consequence of ER-stress signaling and independent of protein glycosylation defects, by inducing ER-stress with thapsigargin, an inhibitor of SERCA calcium pump, or amino-acid limitation that activate the GCN2 dependent pathway (Fig. 2E). Neither thapsigargin or amino-acid limitation reproduced the increment in entosis observed using tunicamycin or NGI-1 in the presence of glucose, therefore, ER-stress was not sufficient to engage the entosis program. Consistently, tunicamycin-induced entosis in the presence of glucose did not require ATF4, as MCF7 cells with an ATF4 Crispr/*Cas*9 knockout for this transcription factor (ATF4^KO^) also demonstrated increased apoptosis+necrosis in the presence of ROCKi as compared to wild-type MCF7 (Fig. 2F).

In the absence of glucose, protein glycosylation would require a continuous flux of carbons from the TCA cycle into the glycolytic pool to fill-up the N-acetyl-glucosamine pathway. Thus, we quantitatively assessed glucose-6-phosphate (G6P) and PEP pools in response to glucose deprivation in wild-type MCF7 cells. The G6P pool was distinctly above background but substantially depleted upon glucose withdrawal (Fig. 2G), whereas PEP levels were significantly higher at the 72 h post-glucose withdrawal point as compared to full glucose conditions, as determined either a biochemical assay (Fig. 2H) or with GC/MS (Supplementary Fig. 1E). Therefore, we hypothesize that the genes responsible for pathways that serve to provide intermediates above PEP in the truncated gluconeogenesis pathway could mediate the flux of carbons that maintained these metabolic pools. In line, we noticed the expression of some of these genes were sensitive to glucose deprivation, namely PCK2 (PEPCK-M) and FBP2 (FBPase) (Fig. 2I). In addition, FBP2 mRNA showed extensive upregulation at several time-points after glucose deprivation, consistent with the upregulation of ATF4 and PCK2 itself.

### Regulation of entosis by pathways other than glycolysis

We [11], and others [12], have shown that, in conditions of nutrient stress, PEPCK-M provides a survival advantage by fluxing carbons into PEP, glycerol-3-phosphate, serine and glycine from alternative substrates, such as glutamine, in addition to upkeeping proline net synthesis, hinting at this pathway’s participation in the regulation of cell growth sustaining processes such as entosis.

**Fig. 3:**
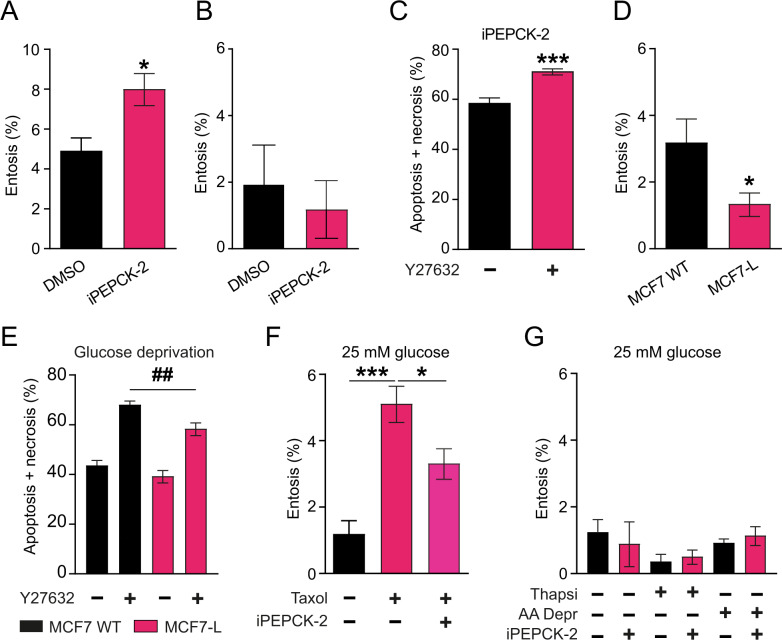
Changes in the activity of the PEPCK-M pathway regulates entosis in the absence of glucose. **A** Percentage of entotic cells observed in wild-type MCF7 cells after 72 h of growth in 0 mM glucose media in the presence of iPEPCK-2 (5 µM) or vehicle (DMSO) (Two-tailed *t*-test; *n* = 5). **B** Percentage of entotic cells observed in wild-type MCF7 cells after 48 h of growth in 25 mM glucose media in the presence of iPEPCK-2 (5 µM) or vehicle (DMSO) (Two-tailed *t*-test; *n* = 3). **C** Percentage of apoptotic+necrotic cells wild-type MCF7 cells detected after 72 h of growth in 0 mM glucose media in the presence of iPEPCK-2 (5 µM), and Y27632 (10 µM) or vehicle (DMSO). Cells were analyzed by flow cytometry using Annexin V-APC and SYTOX™ Green Nucleic Acid Stain Statistical analysis queried Y27632 versus DMSO in the presence of iPEPCK-2 (Two-tailed *t*-test; *n* = 6). **D** Percentage of entotic cells observed in wild-type MCF7 cells and in MCF7 cells overexpressing PEPCK-M (L-PCK2) at the 72 h time point of growth in 0 mM glucose media (Two-tailed *t*-test; *n* = 4). **E** Percentage of apoptotic+necrotic cells wild-type MCF7 and in MCF7 cells overexpressing PEPCK-M (L-PCK2) cells detected after 72 h of growth in 0 mM glucose media in the presence of Y27632 (10 µM) or vehicle (DMSO). Cells were analyzed by flow cytometry using Annexin V-APC and SYTOX™ Green Nucleic Acid Stain Statistical analysis queried MCF7-L versus MCF7 WT (^#^One-way ANOVA with Newman–Keuls post-hoc test; *n* = 8) or Y27632 versus DMSO (*Two-tailed *t*-test; *n* = 8). **F** Percentage of entotic cells observed in wild-type MCF7 cells detected after 24 h of growth in 25 mM glucose media with taxol (1 µM) in the presence of iPEPCK-2 (5 µM) or vehicle (DMSO) (Two-tailed *t*-test; *n* = 4). **G** Percentage of entosis in wild-type MCF7 cells grown in 25 mM glucose media in the presence of thapsigargin (100 nM; 24 h) and amino-acid limitation media (DMEM w/o serine, glycine, arginine and leucine; 48 h). Additionally, cells were treated with iPEPCK-2 (5 µM) or vehicle (DMSO). Statistical analysis queried iPEPCK-2 versus DMSO for each independent treatment; Thapsi and AA Depr (Two-tailed *t*-test; *n* = 8).

Therefore, we aimed to identify whether changes in PEPCK-M activity influence the ability of these cells to undergo entosis using pharmacology (iPEPCK-2) and gain-of-function breast carcinoma cell models (L-PCK2). We quantitated an increased number of vacuole structures indicative of enhanced entotic *cell-in-cell* bodies in the presence of a potent PEPCK-M inhibitor (iPEPCK-2) [13] in wild-type MCF7 cells (Fig. 3A). The effect of PEPCK-M inhibition was specific as iPEPCK-did not change the number of canonical entosis structures in the presence of glucose (Fig. 3B). Unmasking the process with Y27632 (ROCKi) to shift the entotic cells into apoptosis+necrosis showed a significant increase in the number of apoptotic+necrotic cells when PEPCK-M was inhibited with iPEPCK-2 (Fig. 3C). Conversely, gain-of-function stable cultures of MCF7 cells overexpressing PEPCK-M (L-PCK2) reduced the number of entotic body counts (Fig. 3D), and partially abrogated cell death induced by ROCKi as compared to wild-type MCF7 (Fig. 3E). We next examined the effects of PEPCK-M inhibition on an entosis-inducing model independent of metabolic stressors, by stimulating cell-cycle arrest with a mitotic spindle inhibitor, paclitaxel/taxol [14]. Taxol was able to induce entosis in the MCF7 cell model in the presence of glucose as described previously. In this context PEPCK-M inhibitor (iPEPCK-2) did not increase the number of entotic bodies (Fig. 3F), but significantly reduced those entities, an observation that was coupled to increased cell death as determined by Sytox Green/Annexin V analysis, and indicates an interaction between iPEPCK-2 inhibitor and taxol (Supplementary Fig. 1C). These data further suggest that PEPCK-M activity modulation influenced entosis only in the context of glucose limitation. Also, in the presence of glucose, thapsigargin or amino-acid limitation ER-stress together with PEPCK-M inhibition did not significantly change the number of entotic bodies (Fig. 3G).

### PEPCK-M supplies protein glycosylation intermediates and keeps entosis in check

**Fig. 4:**
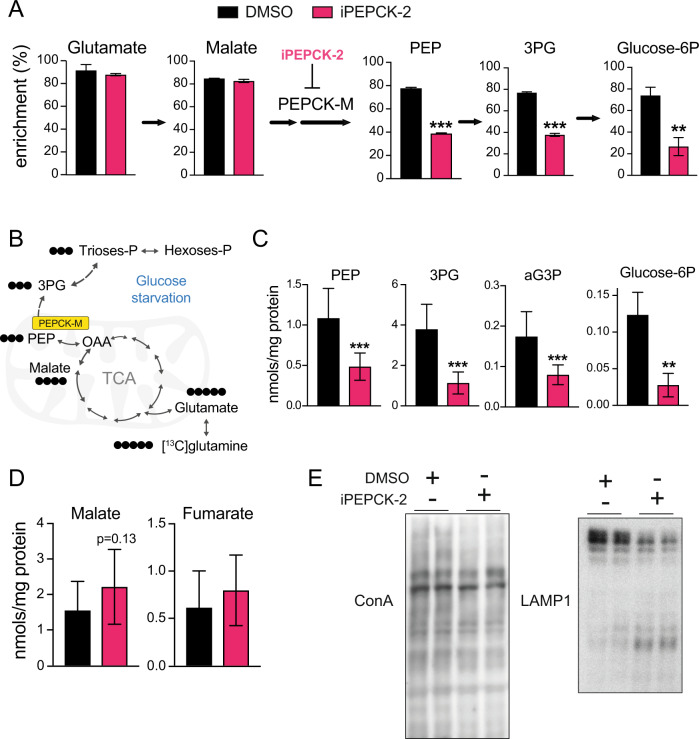
Pharmacological inhibition of PEPCK-M blunts the capacity of the cell to fill-up the glycolytic intermediary pool of metabolites impacting on glycosylation. **A** Enrichment from ^13^C-glutamine into glutamate, malate, phosphoenolpyruvate, 3-phosphoglycerate and glucose-6-phosphate in wild-type MCF7 cells grown in 0 mM glucose media in the presence of iPEPCK-2 (5 µM) or vehicle (DMSO). Metabolites were analyzed using GC/MS spectrometry. (Two-tailed *t*-test; *n* = 3). **B** Scheme of carbon flux from 13C-glutamine into glycolytic pathway. **C** Concentration of selected glycolytic intermediates in wild-type MCF7 cells grown 72 h in 0 mM glucose media in the presence of iPEPCK-2 (5 µM) or vehicle (DMSO). Metabolites were analyzed using GC/MS spectrometry (Two-tailed *t*-test; *n* = 3). **D** Concentration of selected glycolytic intermediates in wild-type MCF7 cells grown 72 h in 0 mM glucose media in the presence of iPEPCK-2 (5 µM) or vehicle (DMSO). Metabolites were analyzed using GC/MS spectrometry (Two-tailed *t*-test; *n* = 3). **E** Western blot for (top panel) Concanavalian A developed proteins, and (bottom panel) human LAMP1 showing full size protein and smeared bands of lower molecular weight representing partially glycosylated protein, after culturing the cells in the absence of glucose in the presence of iPEPCK-2 (5 µM) or vehicle (DMSO).

As shown in Fig. 2H and Supplementary Fig. 1E, PEP levels were unexpectedly high even after glucose deprivation. Therefore, we evaluated whether PEPCK-M quantitatively affects the PEP and the glycolytic intermediary pools from sources other than glucose using iPEPCK-2. GC/MS targeted metabolomics, let us evaluate the contribution of ^13^C-glutamine carbons towards metabolites of the glycolytic pool in conditions of glucose starvation (Fig. 4B). The inhibition of PEPCK impacted significantly on the provision of labeled carbons into PEP and 3-phosphoglycerate (Fig. 4A). Finally, as the presence of FBP1/2 in this model would suggest, we expected the carbon flux from glutamine to incorporate ^13^C-labeled carbons into the upper glycolytic pool, including branching pathways. Indeed, labeled glucose-6-phosphate (Fig. 4A), but also ribose-5-phosphate and ribulose-5-phosphate (Supplementary Fig. 3) were quantitatively identified as products of this metabolic pathway as iPEPCK-2 inhibition obliterated these fluxes in cells under glucose deprivation.

Importantly, the inhibition of the PEPCK-M pathway was selective as there was no effect on the entry of glutamine label, or its incorporation into glutamate or malate (Fig. 4A), consistent with data on PEPCK-M loss-of-function and gain-of-function cervix carcinoma cells (*HeLa*) [11]. The observed contribution of PEPCK-M on glutamine carbon flux towards metabolites of the glycolytic pool was substantial as PEPCK-M inhibition significantly reduced total PEP, 3-phosphoglycerate, glycerol-3-phosphate and glucose-6-phosphate concentrations (Fig. 4C). PEP concentration was also found to be reduced after iPEPCK-2 treatment when assayed biochemically (Supplementary Fig. 1D). Again, changes in the levels of these metabolites above PEP were not the consequence of a TCA cycle pull back as both malate and fumarate did not change significantly after iPEPCK-2 treatment (Fig. 4D). The profound regulation we observed in several metabolite pools by altering PEPCK-M had a consequential bearing on the glycosylation status of total protein in the cell as measured by Concanavalin A binding (Fig. 4E; left panel). The relative amount of intermediate, smeared bands for specific membrane protein markers such as LAMP1 was increased in the presence of iPEPCK-2, indicating defects in glycosylation resulting in the appearance of partially glycosylated peptides are modulated by PEPCK-M activity (Fig. 4E; right panel).

### PEPCK-M is enriched in breast tumors of epithelial origin

**Fig. 5:**
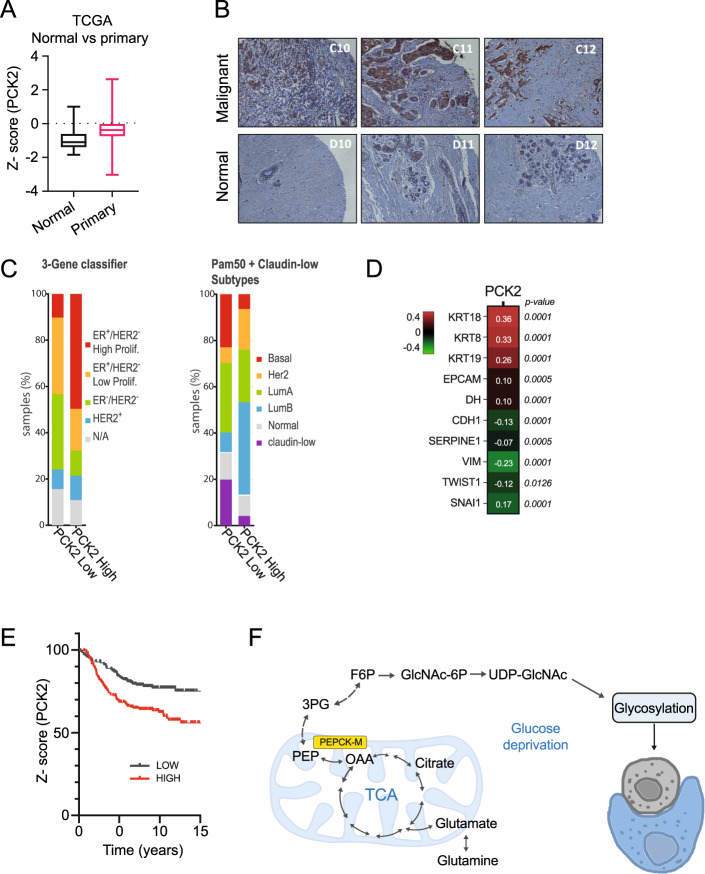
PCK2 coexpresses with breast tumor carcinoma epithelial markers; implications for a role of entosis and PEPCK-M pathway in tumor growth and cancer survival. **A** PCK2 transcript expression on Agilent Gene Chip data obtained from “Normal” (GTEX database) breast tissue and Primary tumors from Yau 2010 study comprising clinical, Agilent Gene Chip gene expression data from datasets GSE2034, GSE5327, GSE7390 and NKI295, TPM units, Agilent normalized gene expression Z-score. Welch’s corrected *t*-test using Xena from UCSC Welch’s *t*-test (*p* = 2.370E-8; *t* = −6.234). **B** PEPCK-M protein content was probed on a tumor microarray panel containing normal and breast carcinoma sections, using a primary antibody against PEPCK-M and developed using DAB peroxidase. **C** PCK2 mRNA expression data from the Metabric human breast carcinoma clinical dataset [15] were ranked at *Z*-score > 1.5 (PCK2 High) and <−1.5 (PCK2 Low) and interrogated for molecular subtype and 3-gene classifier distribution. **D** Kaplan–Meier distant metastasis-free survival probability plot indicating the effect of PCK2 gene expression on survival of tumor patients filtered for clinically relevant marker subtypes (Clusters 2 and 4 from the 306 geneset analysis 16, mostly comprising hormone receptor positive tumors of the Luminal subtype and epithelial lineages). The Median gene expression was used to split samples on High and Low cut-off. Log-rank test was used to calculate hazard ratio (HR), Chi^2^ and significance (Chi^2^ = 11.70, *p* = 0.0006250; HR = 1.921), using built-in statistical package at Xena UCSC. **E** Gene expression correlation matrix between PCK2 and several markers of epithelial (KRT18, KRT8, KRT19, EPCAM) and mesenchymal (SERPINE1, VIM, TWIST1, SMAI1), and E-cadherin (CDH1) preferentially expressed in the epithelial lineage and required for entosis to proceed, and N-cadherin (CDH2), expressed upon a switch to mesenchymal lineage normally involving the corresponding loss of the E-cadherin isoform. Human breast carcinoma clinical samples were queried, and the raw expression data was downloaded prior to statistical analysis from gene expression data comprising datasets GSE2034, GSE5327, GSE7390 and NKI295 [16]. Pearson correlation and *p*-value for each pair set is indicated in the plot. **F** Role of PEPCK-M in the formation of glycosyl moieties for glycosylation of proteins during nutrient stress and the activation of cell-in-cell engulfing.

Entosis is restricted to the epithelial, E-cadherin (CDH1) enriched phenotype [10], therefore, a switch to N-cadherin (CDH2) characteristic of the epithelial-mesenchymal transition would impede the process. Consistent with a role in cancer metabolism and in entosis, PCK2 is overexpressed in breast carcinoma (Fig. 5A, B). In the mammary gland, as in the liver, we observed a switch from PCK1 to PCK2 isoforms going from differentiated tissue to malignant cells [11]. We then examined the distribution of both molecular subtypes and 3-gene classifier signatures in high and low PCK2 expressing tumor samples from breast cancer patients curated in the Metabric dataset [15]. In this analysis, high PCK2 expression samples were enriched in ER + and ER + /Her2- High proliferation subtypes, which also correlated with lower Basal and higher Luminal B/Her2 frequencies (Fig. 5C). Consistently, PCK2 is preferentially enriched in specimens expressing epithelial markers (i.e., KRT18, KRT8, EPCAM, KRT19, CDH1) and negativelycorrelated with the expression of mesenchymal markers (i.e, VIM, TWIST1, SNAI1, SERPINE1, CDH2) (Fig. 5D). Interestingly, PCK2 gene expression has a negative impact on distant metastasis-free survival (Log-rank test Chi^2^ = 11.70, *p* = 0.0006250; HR = 1.921) on a pooled breast cancer microarray dataset [16] filtered for intrinsic molecular subtypes enriched for epithelial markers (dataset comprising Luminal A and B from the PAM50 molecular clusters, or Clusters 2 and 4 from the 306 geneset described in Hu et al. [17]) (Fig. 5E).

## Discussion

Entosis, a form of epithelial cell cannibalism prevalent in human cancers can be triggered by multiple stress signals, including matrix de-adhesion and glucose starvation [1, 2, 3, 19]. The entosis program begins with the formation of epithelial adherens junctions, and the associated generation of actomyosin-contractility, which together drive cell engulfment [18]. However, our understanding on how glucose starvation promotes entosis is very limited, i.e., bioenergetic collapse, but also downstream activation of stress signals secondary to glycosylation defects might play a relevant role [19].

For example, in a different model, where de-adhesion triggers E-cadherin migration and entosis [10], certain mutants of E-cadherin which are constitutively non-glycosylated can induce this process in the absence of detachment, suggesting that glycosylation defects in E-cadherin potentiate their capacity to stimulate alterations in cell structure and motility prior to the induction of entosis [20–22]. Therefore, protein glycosylation per se might be responsible for the initiation of entosis. We tested this hypothesis and indeed, found that glucose deprivation resulted in reduced total protein N-glycosylation, and increased partially glycosylated LAMP1 and E-cadherin band smears, in parallel with increased entosis. N-acetyl-glucosamine (NAG), a non-metabolizable donor of glycosyl moieties for protein glycosylation [23] partially rescued this process and inhibited entosis derived cell death. Importantly, in glucose fed cells, treatment with tunicamycin or NGI-1, inhibitors of protein N-glycosylation, increased entosis, as quantitated directly under the microscope and indirectly by measuring apoptosis+necrosis in the presence of ROCKi. Importantly, ATF4 was not required for tunicamycin/NGI-1 induced entosis in the presence of glucose as cells with a Crispr/*Cas*9 deletion of ATF4 (ATF4^KO^) increased apoptosis+necrosis population in the presence of ROCKi. Likewise, thapsigargin or amino-acid limitation treatments that induce a canonical ER-stress response did not mimic the effect of tunicamycin/NGI-1. These data were consistent with prior results showing that the activation of ER-stress via GCN2 after amino-acid limitation had limited effects on entosis [19].

Hence, we turned out attention to pathways that might be responsible for the provision of carbons into N-glycosyl moieties in the cell as they might have a role in the regulation of the process of entosis in this model. One such pathway is the gluconeogenic flux of carbons from TCA cycle intermediaries into the glycolytic pool. As a member of the PEPCK family with a role in gluconeogenesis [5] (GTP; EC 4.1.1.32), this enzyme catalyzes the GTP dependent conversion of oxaloacetate (OAA) to phosphoenolpyruvate (PEP), and unlike its cytosolic counterpart, PEPCK-C, PEPCK-M is widely expressed (i.e., T- and B-cells, pancreatic β-cells, liver, neurons, and undifferentiated tissues such as embryonic stem cells and tumors). In the absence of glucose, PEPCK-M gene expression (PCK2) is positively regulated by ATF4 and its role in coping with nutritional stress in tumor cells has been well described and is consistent with the ability of some cancer cell lines, for example non-small cell lung cancer (NSCLC) cells, to withstand glucose limitation, and even proliferate in the presence of other carbon sources such as glutamine [24]. We, therefore, asked whether PEPCK-M might regulate entosis, and further if entosis is masking a more general role for PEPCK-M in the regulation of cell proteostasis and viability.

PEPCK-M inhibition resulted in increased number of engulfed bodies under the microscope and higher apoptosis+necrosis population after treatment with Rho-GTPase kinase (ROCK) inhibitor Y27632 (ROCKi). In contrast, PEPCK-M overexpression was inhibitory, demonstrating a clear role for PEPCK-M on the regulation of entosis. Furthermore, iPEPCK-2 treatment or PEPCK-M overexpression did not alter ER-stress signaling and inhibiting PEPCK-M in the absence of ATF4 (ATF4^KO^) did not ablate the stimulation of entosis observed with PEPCK-M inhibition (data not shown), suggesting that PEPCK-M regulation of entosis was independent of its interaction with the ER-stress pathway.

Hence, a direct role for PEPCK-M in glycosyl moiety formation or maintenance shown here in basal, glucose deprived conditions are in agreement with neuroprogenitor cell studies where PEPCK-M had a role in the shift to the progenitor/stem phenotype, showed that inhibition of this pathway resulted in reduced production of highly glycosylated extracellular matrix laminin [25]. The participation of PEPCK-M in the *de novo* formation of N-acetyl-glucosamine moieties would reconcile all the data above, therefore we examined both the glycolytic intermediary pool and the direct fluxes that feed into them using ^13^C-labeled glutamine. A marked reduction of PEP and upstream metabolites upon PEPCK-M inhibition was noticed in MCF7 cells. Moreover, the flux of labeled carbons from ^13^C-glutamine was also identified and almost exclusively due to PEPCK-M as iPEPCK-2 treatment blocked 90% of the specific label enrichment above PEP. However, preliminary biochemical data after overexpression of PEPCK-M showed no increased PEP (data not shown), probably as wild-type MCF7 cells already express a very substantial amount of PEPCK-M protein, and the pathway is very active as demonstrated by increased PEP concentration upon glucose starvation. In other models, such as HCT116, SW480 and HeLa, PEP concentration is consistently lower upon glucose removal, probably because these cells express lower levels of endogenous protein [26]. Consistently, PEPCK-M overexpression in these other models increased the PEP pool [11, 26], suggesting metabolic flux control. These data fit with the expected contribution of carbons other than glucose to the various biosynthetic pathways branching out from the glycolysis pool in a PEPCK-M dependent manner [11, 12, 24]. Unfortunately, a confirmation of PEPCK-M dependent labeling of glutamine carbon into UDP-N-acetyl-glucosamine was not possible since we could not identify this metabolite or its precursor, N-acetyl-glucosamine, in the spectra obtained at the GC/MS due to technical limitations with this technique. However, the capacity of PEPCK-M activity to fill-up all of the glycolytic metabolite pool above PEP conclusively substantiates the contribution of this pathway towards the synthesis of all glycosyl moieities, as expected from previous studies in the liver [5].

In support of this hypothesis, we observed the presence of the fructose-bisphosphatase (FBP1 and FBP2) in these cells, as mRNA content of both isoforms were quantitated, being the expression of FBP2 very low but sensitive to glucose deprivation. FBP1/2 was also significantly co-expressing with PCK2 in tumor samples subset analyzed in Fig. 5D (data not shown). This is also consistent with the view that MCF7 cells produce glycogen at early phases of hypoxia due to enhanced expression of GYS1 that can be later utilized upon PYGL expression when exposed to long-term hypoxia stimuli [27]. However, glycogen stores might not represent a sizable source of glucose carbon in conditions of long-term glucose deprivation. Furthermore, MCF7 cells investigated here showed no malic enzyme flux activity in the metabolomics data, as we found no pyruvate labeling from ^13^C-glutamine in the presence of iPEPCK-2 inhibitor (Supplementary Fig. 1E), hence, in conditions of limited glucose supply, pyruvate formation was all dependent on PEP generated via PEPCK-M (*see* Supplementary Fig. 3 for full TCA-cycle enrichment data).

In this study, interfering with protein glycosylation by inhibiting PEPCK-M increases entosis rates, whereas restoring/ensuring protein glycosylation by overexpressing PEPCK-M stalls entosis. However, the role of entosis in the tumor has been controversial [28]. Entosis is commonly pictured as a cell survival mechanism, although this interpretation has been recently challenged as the number of *CiCs* in breast carcinoma specimens associated with better prognosis in patients of the Luminal B subtype [29], mostly of the epithelial lineage. Similar conclusions were drawn by Wang et al. [30] in human esophageal squamous cell carcinoma, indicating that entosis might encompass an additional layer of the complex cell death machinery in tumors. Enrichment of PCK2 in tumors expressing epithelial markers, and its negative impact on distant metastasis-free survival on an epithelial filtered dataset described here puts weight on the later hypothesis as PEPCK-M activity would favor tumor cell survival while inhibiting entosis. One possible scenario would include the capacity of PEPCK-M in this model to maintain high levels of PEP upon glucose removal which in turn can trigger Ca^2+^ signaling and pro-survival pathways such as NFAT and c-Myc through its inhibition of SERCA, as described by us [26] and others [31].

In summary, as illustrated in Fig. 5F, the present work describes the mechanisms implicated in the regulation of entosis by PEPCK-M through protein glycosylation and suggests a unifying mechanism for the convergence of several hallmarks of cancer cells, including anchorage independence and metabolic stress, on cell cannibalism, a frequent phenomenon in tumors. Alternatively, PEPCK-M might directly impact on proteostatic metabolites (i.e., serine/glycine synthesis pathway; alanine supply in the absence of malic enzyme) or the regulation of cell signaling through the capacity of PEP to influence Ca^2+^ fluxes [26] as mechanisms upstream of the disturbances described here. Regardless of the precise mechanism, these data provide support for the proposition that PEPCK-M enabling entosis, and other pathways involved in coping with nutrient/environmental stress, have a significant contribution to the clinical outcome of these tumors.

## Supplementary information

Legends to Supplementary Figures

Supplemental Fig. 1

Supplemental Fig. 2

Supplemental Fig. 3

Original WB Fig. 1F ecadh

Original WB Suppl Fig. 1B pEIF2a

Original WB Suppl Fig. 1A ATF4

Original WB Suppl Fig. 1A ATF4 Overlap

Original WB Suppl Fig. 1B pEIF2a Overlap

Original WB Ecadherin

Original WB Ecadherin Overlap

Checklist

## Data Availability

There are no restrictions on material availability or other relevant information/data upon request to jperales@ub.edu.
