# Supplementary material for: Glycosylation defects, offset by PEPCK-M, drive entosis in breast carcinoma cells
Source: Cell Death Dis. 2022 Aug 24;13(8):730. doi: 10.1038/s41419-022-05177-x (PMC9402552; doi:10.1038/s41419-022-05177-x)
Supplement: Supplementary file 1 — Legends to Supplementary Figures [file 41419_2022_5177_MOESM1_ESM.docx]

**SUPPLEMENTARY FIGURE 1*:***

1. Western blot analysis of ATF4 protein after culturing the cells in several conditions inducing stress. Wild-type MCF7 cells were grown in 25 mM glucose media in the presence of vehicle (CT, DMSO), tunicamycin (TUN, 3 µg/ml, 48h), NGI-1 (10 µM, 48h), thapsigargin (100 nM; 24h) or amino-acid limitation media (AA Depr, DMEM media w/o serine, glycin, arginin and leucine; 48h).
2. Western blot for human LAMP1, p-eIF2α, E-cadherin and gamma tubulin after culturing the cells in the 25 mM glucose media in the presence of tunicamycin (TUN, 3 µg/ml, 48h) or vehicle (DMSO).
3. Percentage of apoptotic+necrotic cells wild-type MCF7 detected after 24h of growth in 25 mM glucose media in the presence of Taxol (1 µM) and treated with iPEPCK-2 (5 µM) or vehicle (DMSO). Cells were analyzed by flow cytometry using Annexin V-APC and SYTOX™ Green Nucleic Acid Stain. Statistical analysis queried Taxol treatment versus DMSO in the presence of iPEPCK-2 (Two-tailed *t*-test; n=3). (n=6).
4. Concentration of PEP in wild-type MCF7 cell extracts after 24h of growth in 0 mM glucose media in the presence of iPEPCK-2 (5 µM) or vehicle (DMSO). Concentration was measured by using enzymatic assay and values were normalized by protein content (Two-tailed *t*-test; n=3).
5. Concentration of pyruvate, citrate and PEP in wild-type MCF7 cells grown in the presence of 25 mM glucose or 0 mM glucose. Concentration of metabolites was analyzed using GC/MS spectrometry (Two-tailed *t*-test; n=3).

**SUPPLEMENTARY FIGURE 2*:***

1. TCA-cycle metabolite isotopologues distribution after 72h growth in 0 mM glucose media, where for last 9h of experiment, U-^13^C labeled glutamine was added. Metabolites were analyzed using GC/MS spectrometry (Two-tailed *t*-test; n=3).

**SUPPLEMENTARY FIGURE 3*:***

1. Total enrichment of TCA-cycle metabolites after 72h growth in 0 mM glucose media, where for last 48h of experiment, U-^13^C labeled glutamine was added. Metabolites were analyzed using GC/MS spectrometry (Two-tailed *t*-test; n=3).
