# Supplementary figures and images for: Glycosylation defects, offset by PEPCK-M, drive entosis in breast carcinoma cells
Source: Cell Death Dis. 2022 Aug 24;13(8):730. doi: 10.1038/s41419-022-05177-x (PMC9402552; doi:10.1038/s41419-022-05177-x)

Figure S1

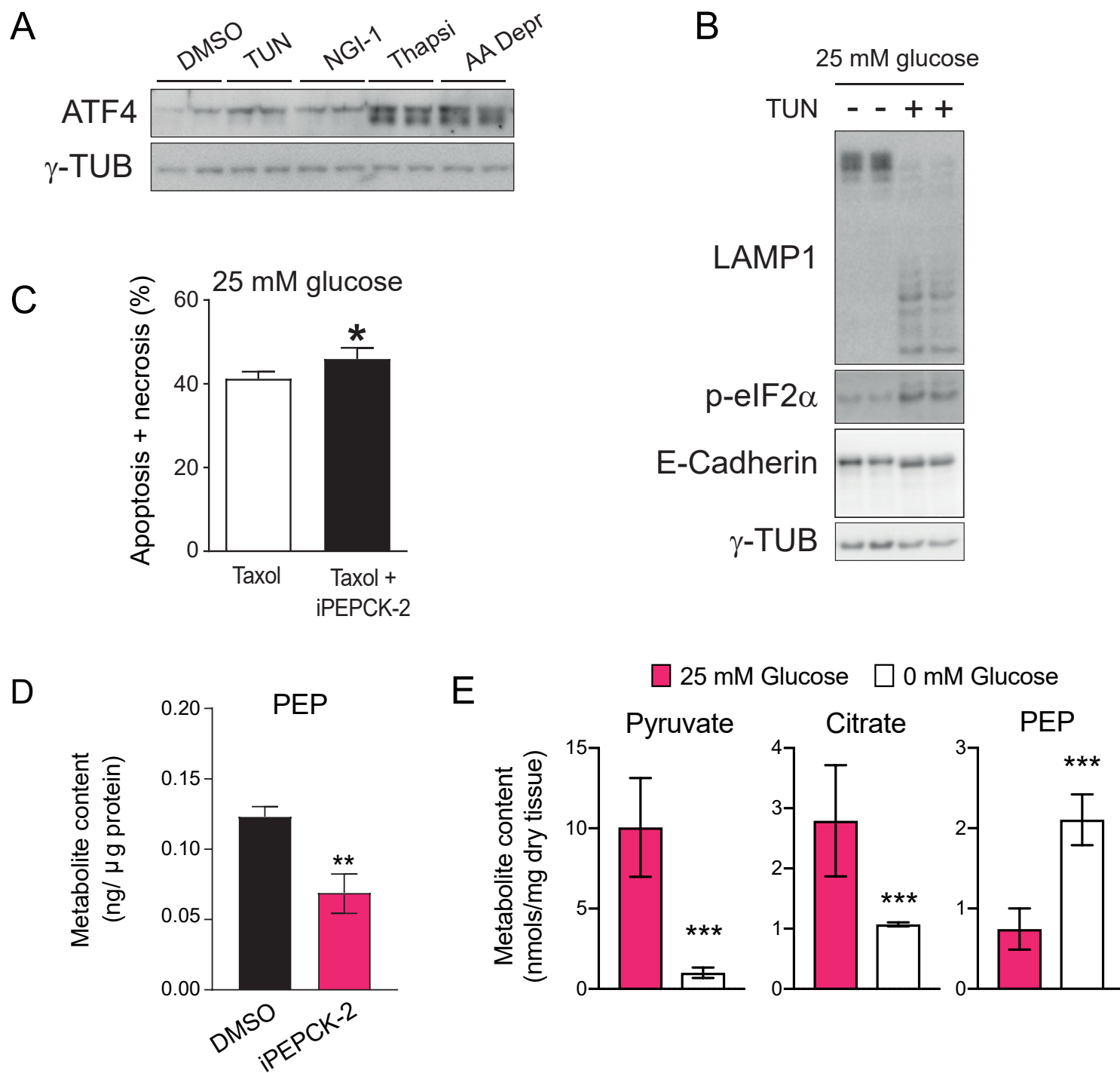

Supplement: Supplementary file 2 — Supplemental Fig. 1 [file 41419_2022_5177_MOESM2_ESM.pdf]

Figure S2

A

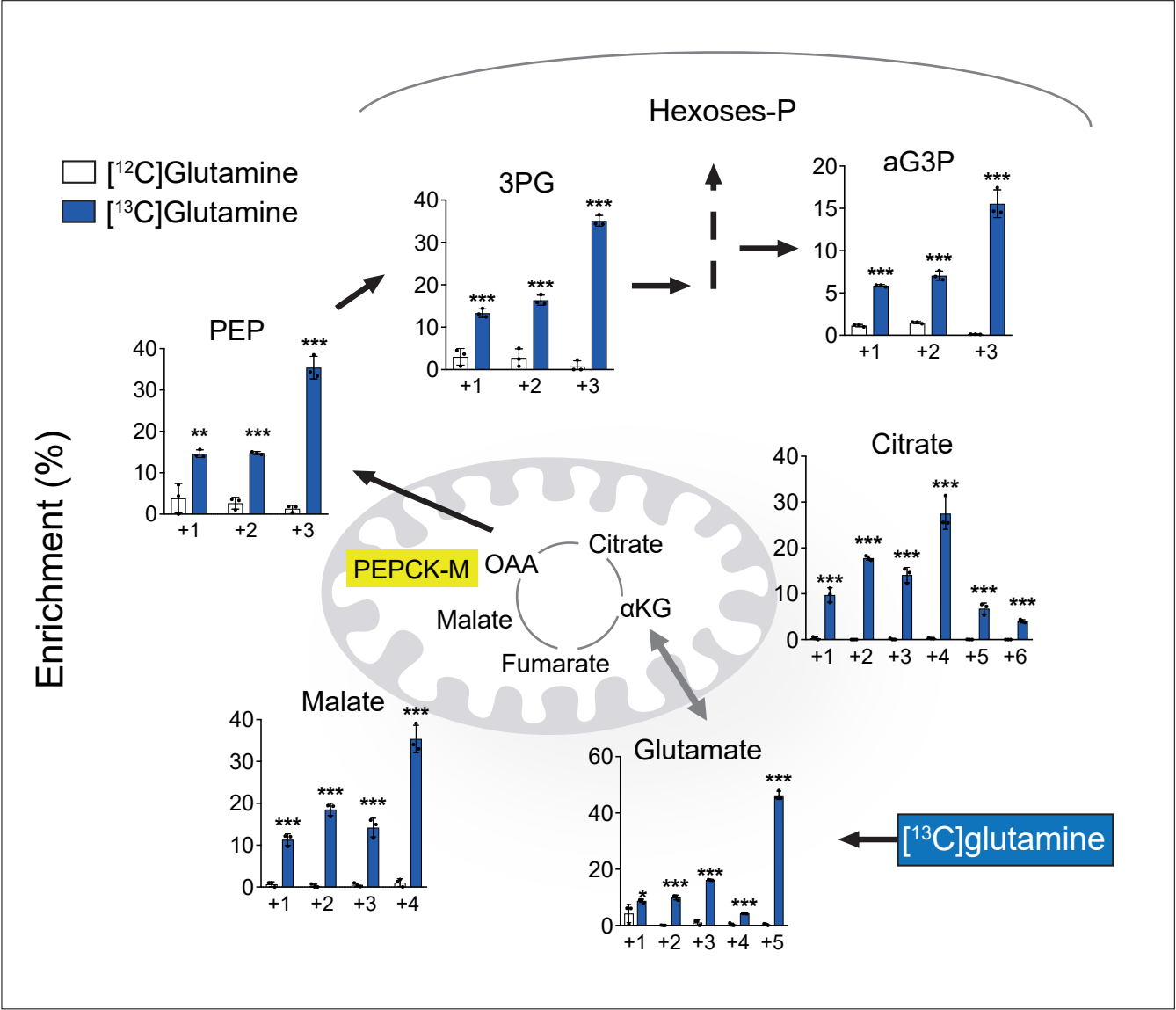

Supplement: Supplementary file 3 — Supplemental Fig. 2 [file 41419_2022_5177_MOESM3_ESM.pdf]

Figure S3

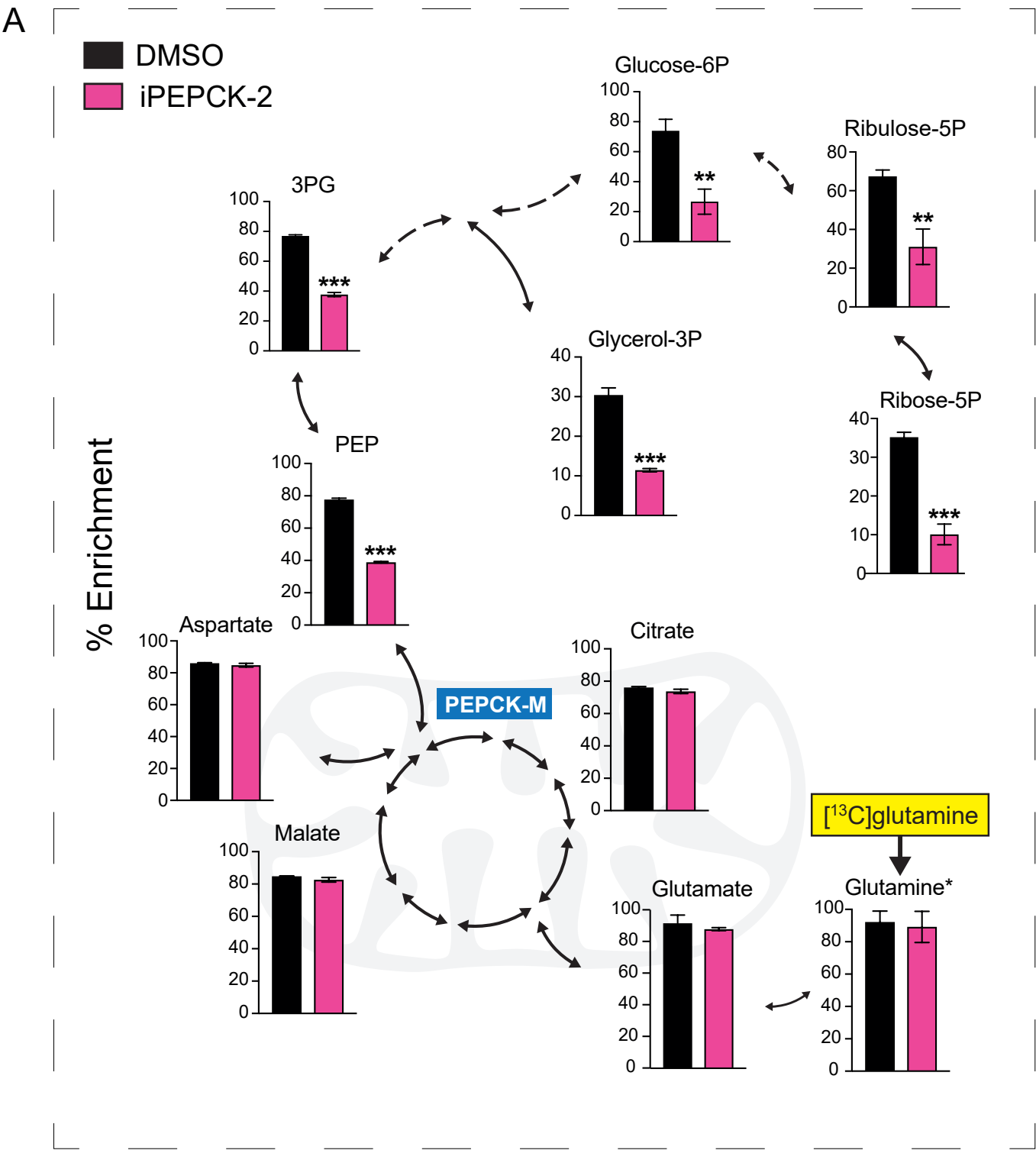

Supplement: Supplementary file 4 — Supplemental Fig. 3 [file 41419_2022_5177_MOESM4_ESM.pdf]

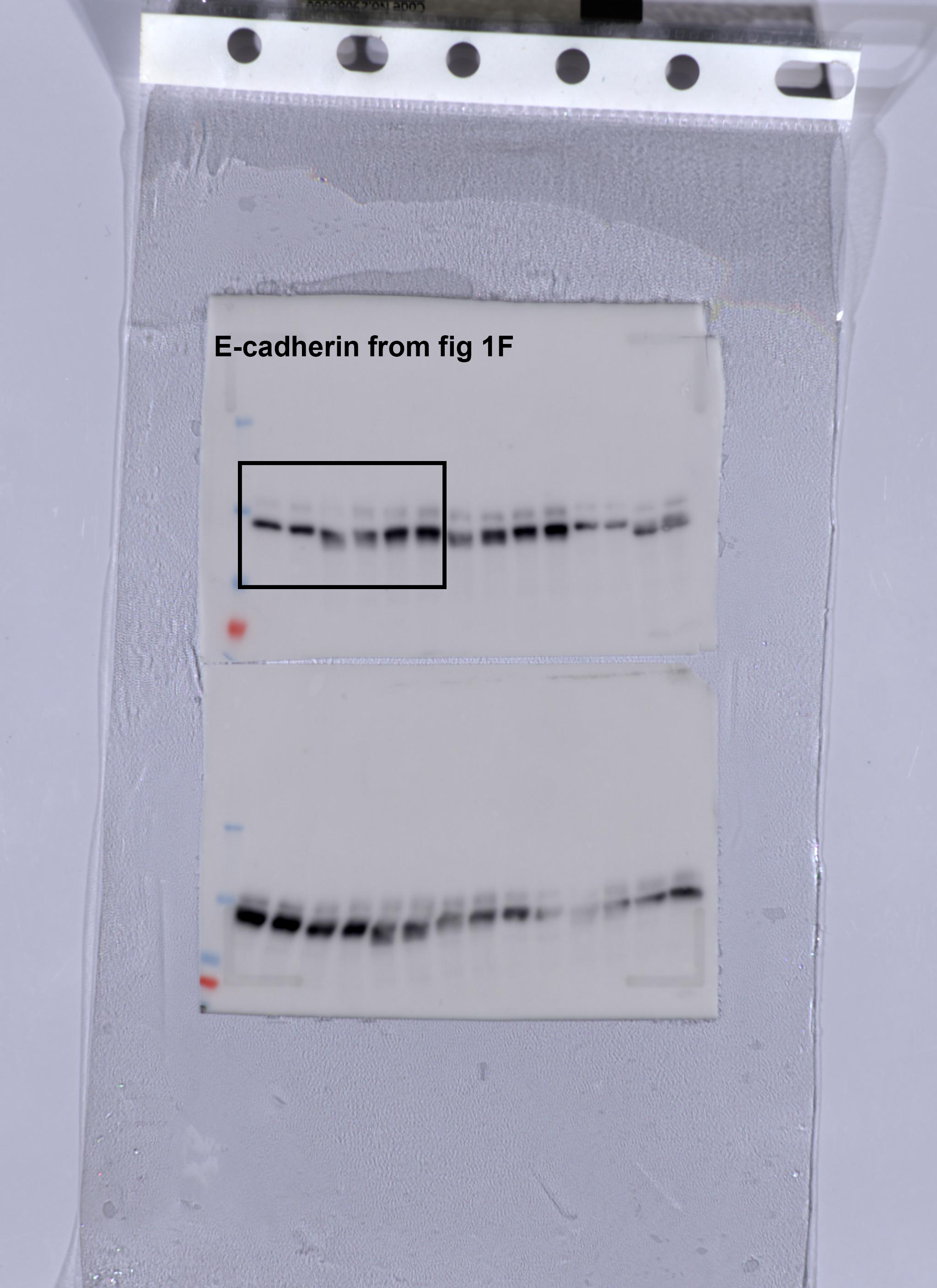

Supplement: Supplementary file 5 — Original WB Fig. 1F ecadh [file 41419_2022_5177_MOESM5_ESM.tif]

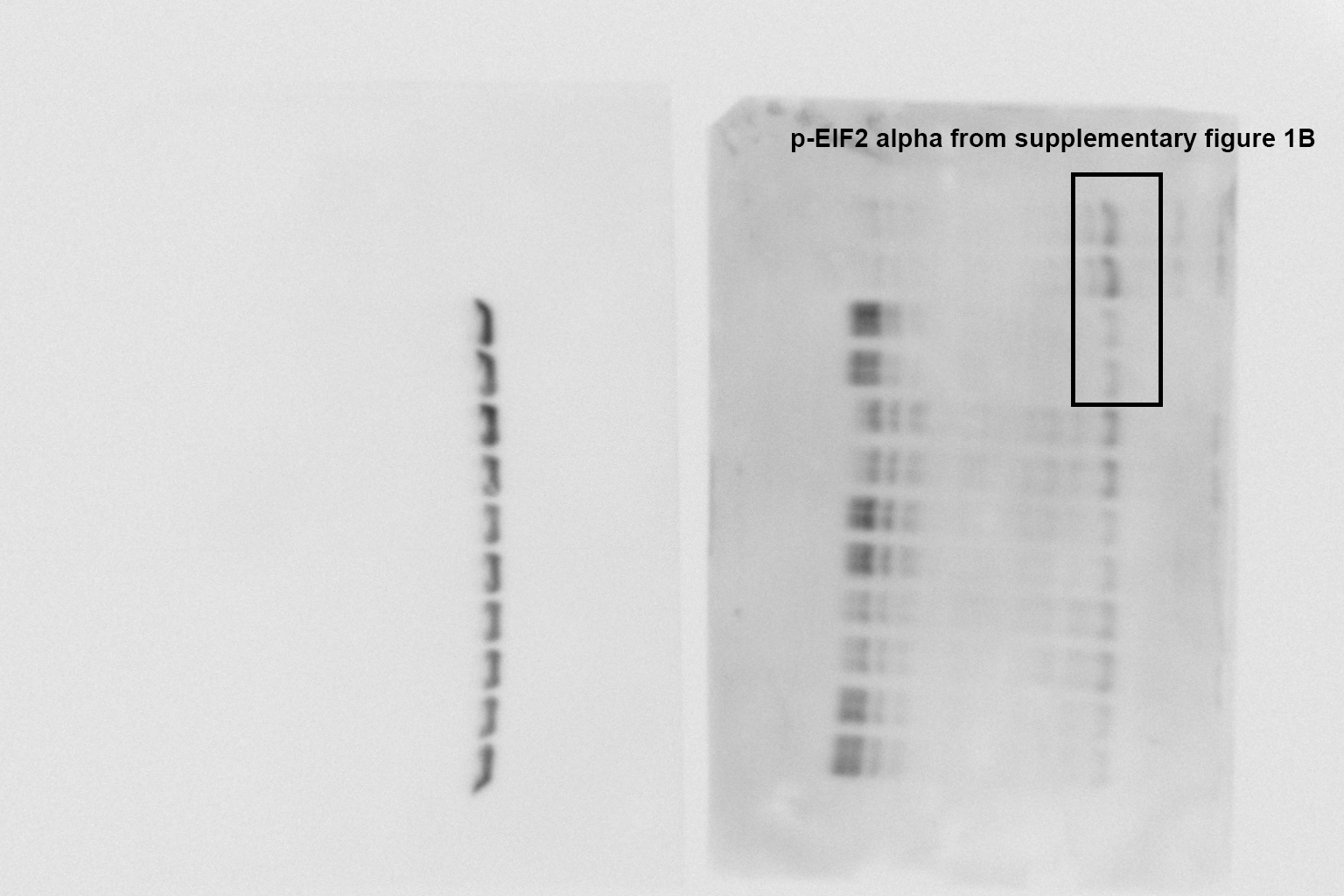

Supplement: Supplementary file 6 — Original WB Suppl Fig. 1B pEIF2a [file 41419_2022_5177_MOESM6_ESM.tif]

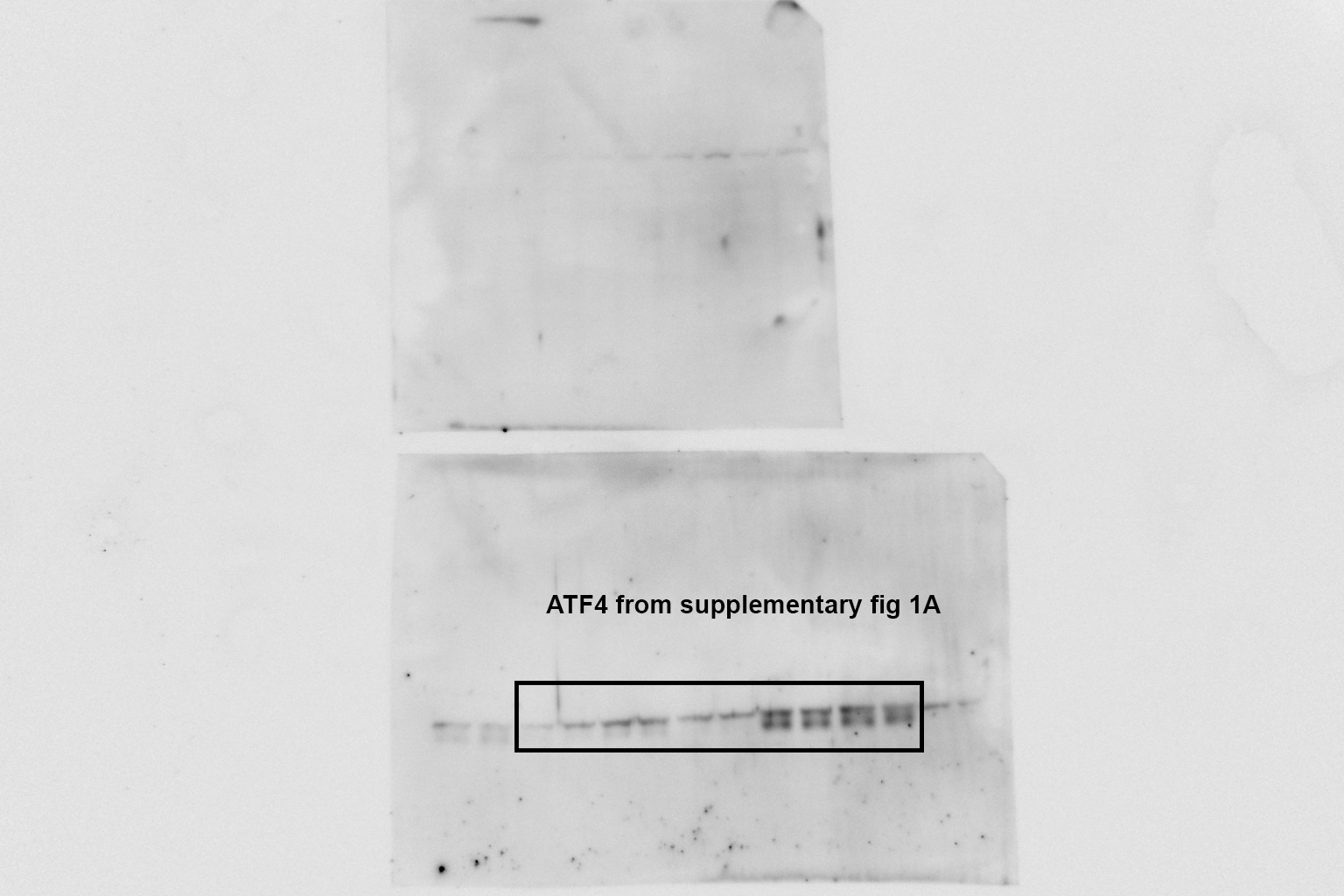

Supplement: Supplementary file 7 — Original WB Suppl Fig. 1A ATF4 [file 41419_2022_5177_MOESM7_ESM.tif]

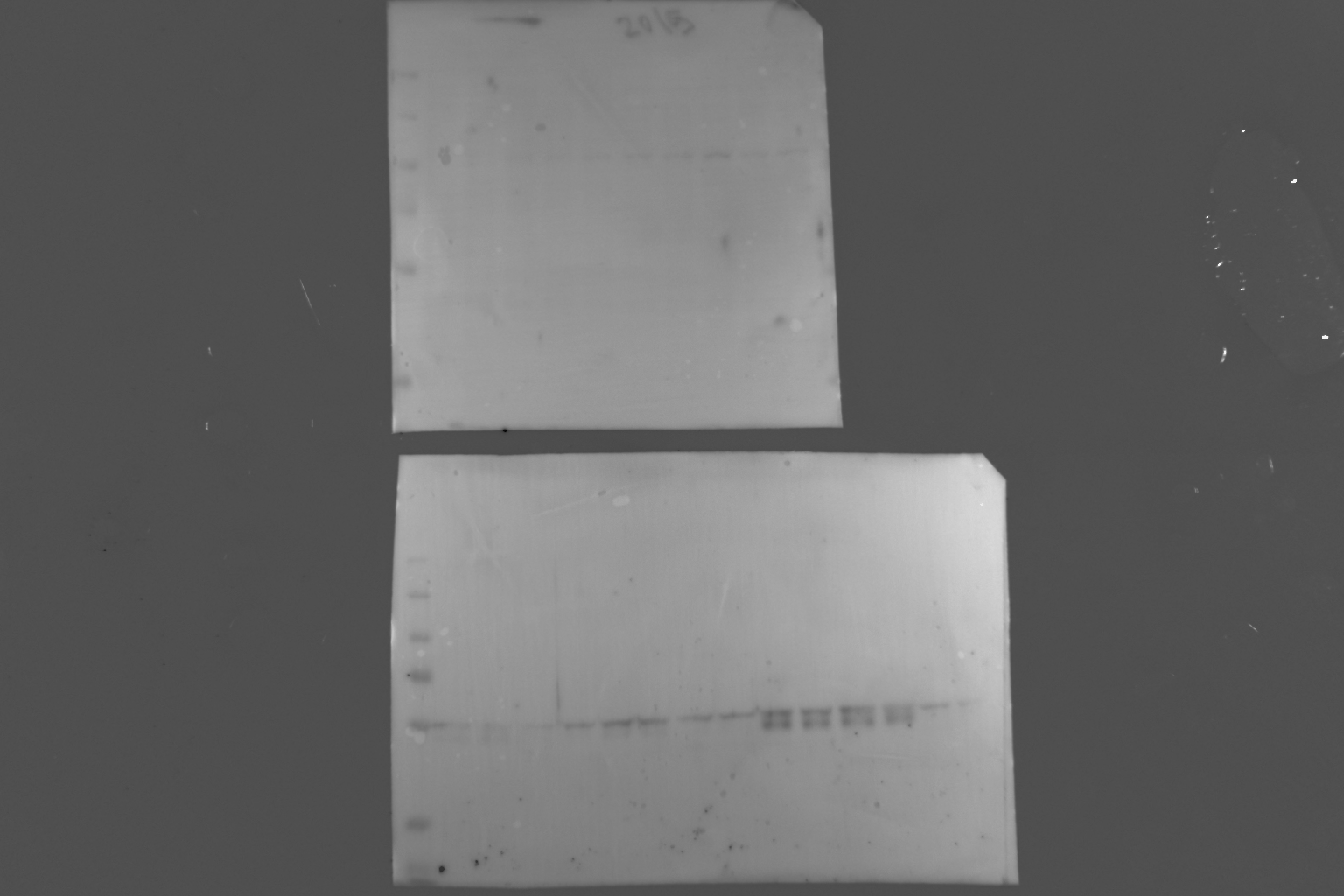

Supplement: Supplementary file 8 — Original WB Suppl Fig. 1A ATF4 Overlap [file 41419_2022_5177_MOESM8_ESM.tif]

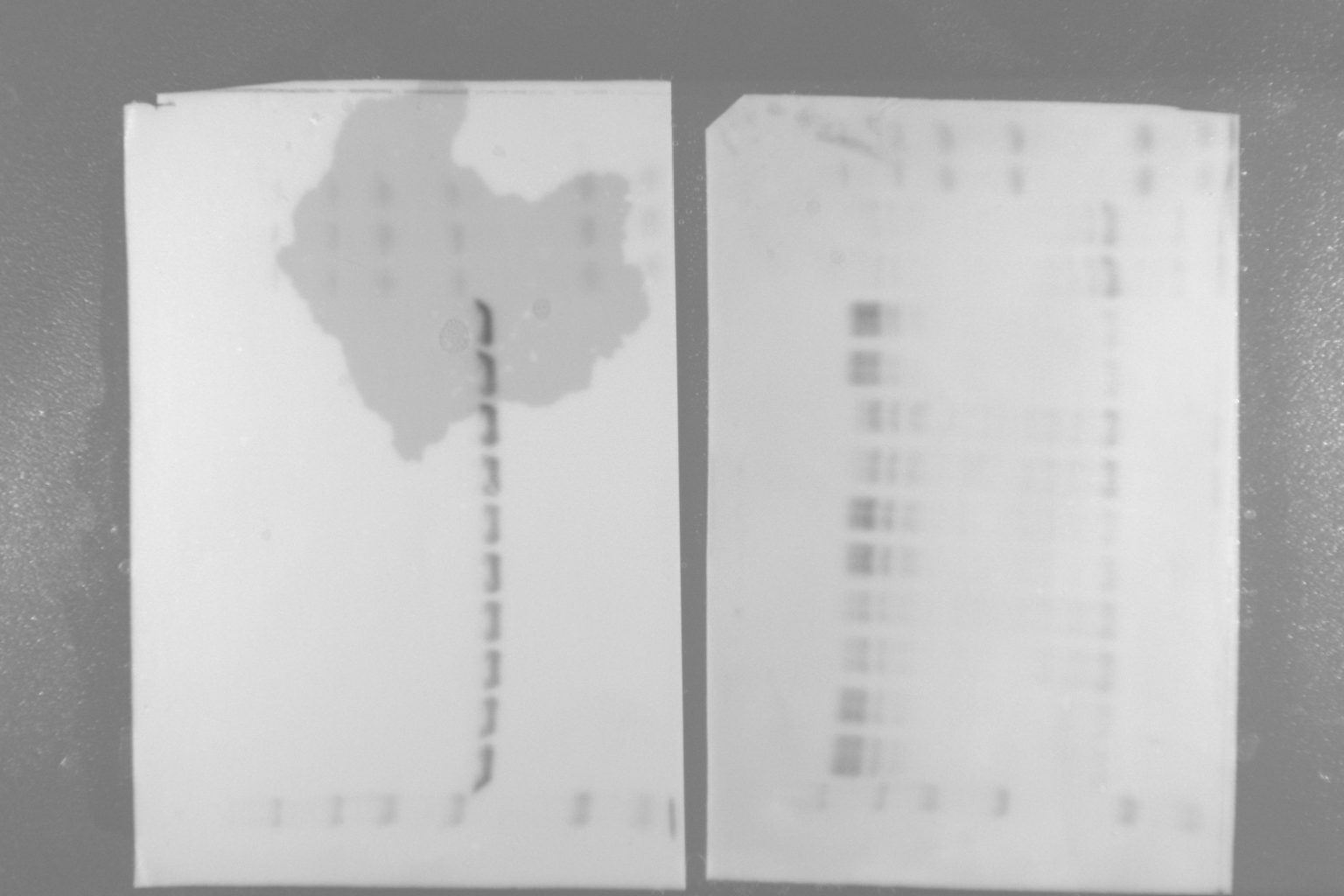

Supplement: Supplementary file 9 — Original WB Suppl Fig. 1B pEIF2a Overlap [file 41419_2022_5177_MOESM9_ESM.tif]

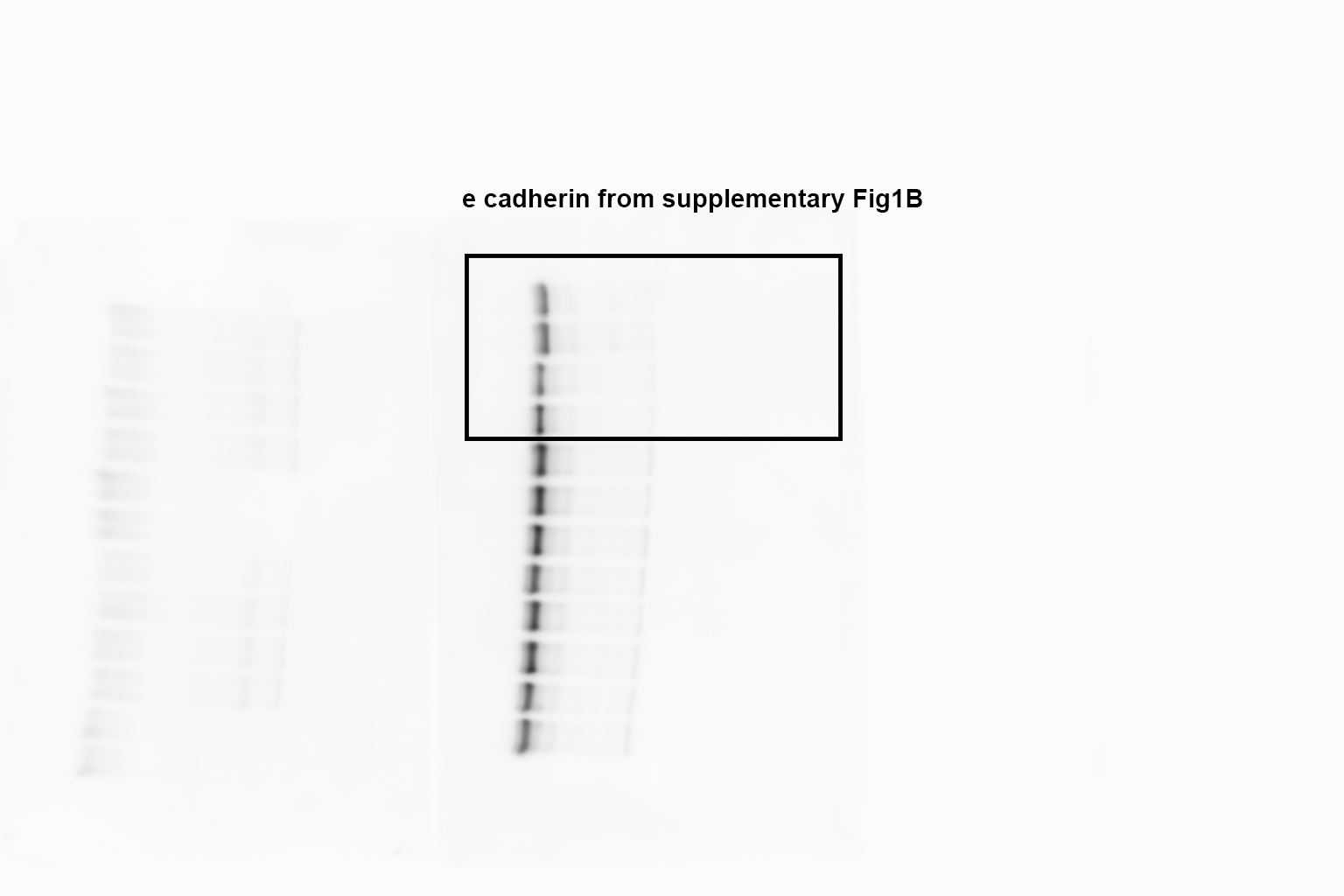

Supplement: Supplementary file 10 — Original WB Ecadherin [file 41419_2022_5177_MOESM10_ESM.tif]

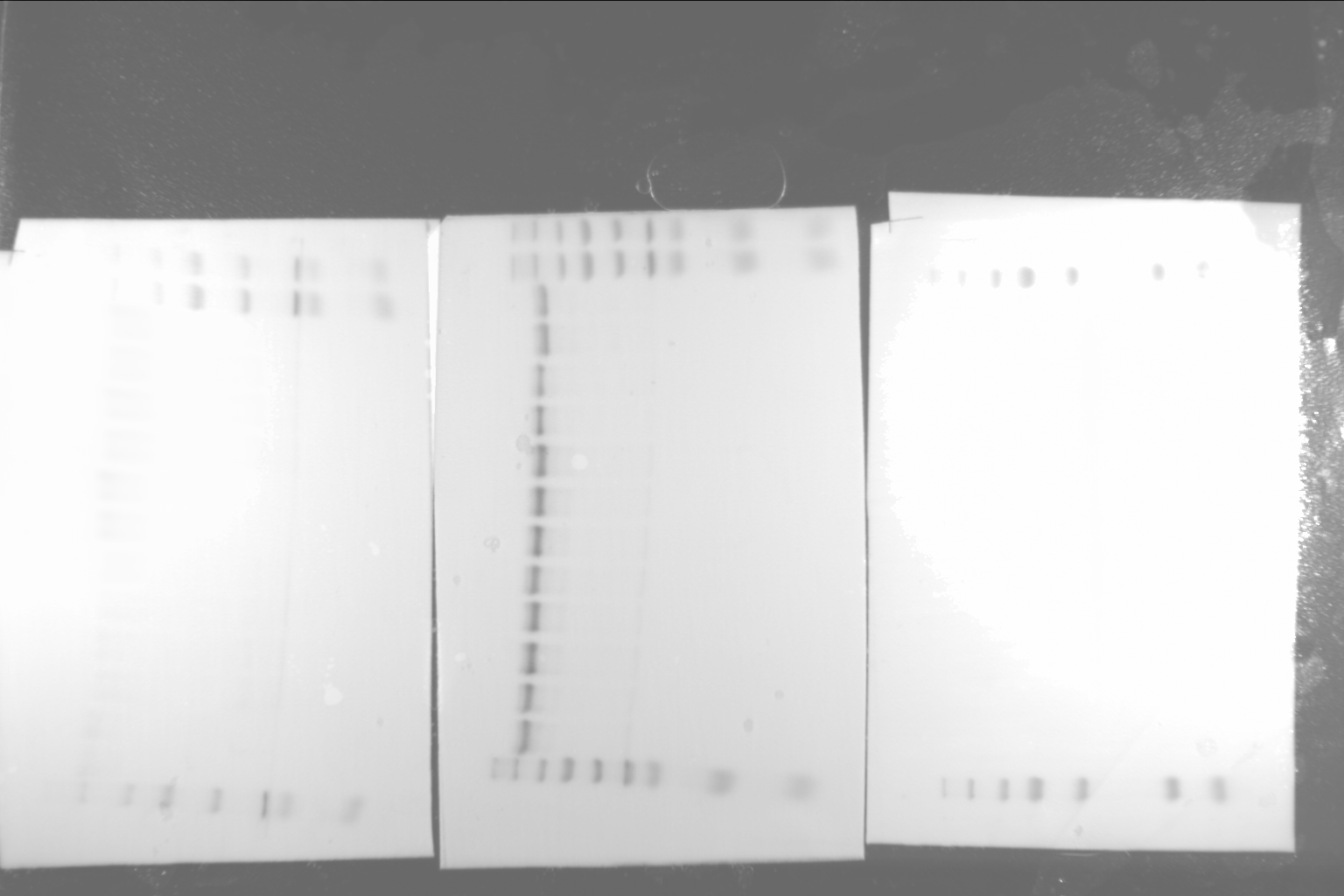

Supplement: Supplementary file 11 — Original WB Ecadherin Overlap [file 41419_2022_5177_MOESM11_ESM.tif]
